# Effects of intrapulmonary viral tropism and cytokine expression on the histological patterns of cytomegalovirus pneumonia

**DOI:** 10.1111/j.1440-1827.2012.02849.x

**Published:** 2012-08-28

**Authors:** Yoshifumi Arai, Takashi Tsuchida, Isao Kosugi, Hideya Kawasaki, Shiori Meguro, Mana Kinoshita, Satoshi Baba, Matsuyoshi Maeda, Yuichiro Shinmura, Yoshihiro Tsutsui, Toshihide Iwashita

**Affiliations:** 1 Department of Pathology, Hamamatsu University School of MedicineHamamatsu, Japan; 2 Hospital Pathology Unit, Central Clinical Facility, Hamamatsu University HospitalHamamatsu, Japan; 3 Faculty of Health Sciences, Hamamatsu UniversityHamamatsu, Japan; 4 Division of Pathology, Clinical Laboratory, Toyohashi Municipal HospitalToyohashi, Japan; 5 Division of Pathology, Clinical Laboratory, Kakegawa Municipal General HospitalKakegawa, Japan; 6 Department of Pathology, Saitama Medical University International Medical CenterHidaka, Japan

**Keywords:** chromogenic *in situ* hybridization, cytomegalovirus pneumonia, double immunostain, integrin β6, interleukin-8, transforming growth factor-β1

## Abstract

Pulmonary cytomegalovirus (CMV) infection causes fatal CMV pneumonia (CMVp) in Immunocompromised patients; however, the mechanisms underlying CMV-Infection-Induced pulmonary lesion development remain largely unknown. We examined the relationship between CMVp patterns and Intrapulmonary viral tropism, Including expression of Inflammatory cytokines and related molecules. Double Immunohistochemistry of CMV antigen and cellular markers showed that epithelial tropism was associated with a diffuse alveolar damage (DAD) pattern (CMVp-DAD) while stromal tropism was associated with a predominantly interstitial inflammation/fibrosis (IIF) (CMVp-IIF) or a combination of DAD and IIF (CMVp-complex). Transforming growth factor (TGF)-β1 expression was relevant to CMV-induced tissue injury, and its expression was higher in CMVp-complex and CMVp-IIF than in CMVp-DAD. Expression of integrin β6 (ITGB6), an adhesion molecule and important activator of TGF-β1 in interstitial pneumonia, was lost in CMV-infected pneumocytes, especially CMVp-DAD, whereas CMV-negative pneumocytes in CMVp-complex and CMVp-IIF showed overexpression. Diffuse *interleukin (IL)-8* up-regulation and strong expression were present in both CMV-infected pneumocytes and stromal cells only in CMVp-IIF cases with marked interstitial neutrophilic infiltration. On the basis of viral tropism and the expression of TGF-β1, ITGB6, and *IL-8*, we conclude that CMV-Infected pulmonary cells play an Important role in the development of diverse CMVp patterns.

Cytomegalovirus (CMV) is a major pathogenic microbe in immunocompromised individuals, and CMV pneumonia (CMVp) is a critical complication because of the high fatality.^1^^,^^2^ The clinical findings of CMVp have been well documented;^3^^,^^4^ however, how CMV infection causes pulmonary lesions is not yet well understood.

Cytomegalovirus pneumonia, a secondary interstitial pneumonia (IP), exhibits various histopathological characteristics, i.e., focal or diffuse interstitial lesions with/without hemorrhage, hyaline membrane, and necrobiosis.^5–7^ It has been reported that CMV infects a wide variety of cell types, including pneumocytes, fibroblasts, macrophages, and endothelial cells in the lung.^8^ Although cytomegalic cells are a well-known hallmark of CMV-infected cells, only a few reports have immunohistochemically confirmed these cell types.^8–10^ Moreover, to our knowledge, the proportions of CMV-infected cell types among the diverse patterns of CMVp have not been described in previous reports.

Many growth factors and cytokines have been identified as pathogenetic factors for the initiation or progression of both idiopathic and secondary IP.^11^^,^^12^ Transforming growth factor (TGF)-β1 has been implicated as a pivotal molecule in the development of acute and chronic IP^13^^,^^14^ and CMVp.^15^ CMV infection was reported to induce production of TGF-β1,^16^ and this protein also enhanced viral replication in some CMV-infected cells.^17^ Inflammatory cytokines, such as tumor necrosis factor-α, interleukin (IL)-6, and IL-8, were highly expressed in IP,^18–20^ and were up-regulated by CMV infection.^21,22^ Furthermore, high expression of IL-8 and its receptor in CMV-infected human lung fibroblasts enhanced its function in an autocrine manner and promoted CMV replication *in vitro*.^23^ Therefore, CMV-infected cells may play an important role in the formation of IP lesions in CMVp.

However, these findings may not be sufficient to account for the diverse histology of CMVp. Very few reports have analyzed CMVp pathogenesis from a histopathological point of view.^8^^,^^24^ In this study, we focus on the types of CMV-infected cells and the expression of inflammatory cytokines and related molecules in the various histological types of CMVp.

## MATERIALS AND METHODS

### Case selection

From the autopsy files of the Department of Pathology, Hamamatsu University School of Medicine, 12 cases of severe CMVp, with an average of at least 100 CMV antigen-positive cells in each immunohistochemistry (IHC) section, were selected (Table 1) to examine the characteristic CMV-induced changes. Cases of mild CMVp with fewer CMV-positive cells, as well as cases concomitant with other opportunistic infections such as pneumocystis, fungi, severe bronchopneumonia, or intrapulmonary neoplasms were excluded. Two cases of idiopathic pulmonary fibrosis (IPF) (case nos. 9 and 12) were included; however, the remarkably CMV-infected lesions were carefully compared with the non-CMV-infected areas.

**Table 1:** Clinical data and histopathological findings of 12 autopsy cases

Case no.	Age (yr)	Sex	Primary disease and complications	Pulmonary lesions††	Duration (wk)‡‡	Other CMV-infected organs
1	41	W	Systemic lupus erythematosis†	DAD, (PE, PH)	2	Pnc, UGI, Kdn, LGI, Adr, Thy, Ovr, Utr, Liv, Spl, Gbl, Hrt,
2	69	W	Systemic lupus erythematosis†	DAD, (BP, PE)	1	Pnc, UGI, Kdn, LGI, Liv, Thy, Ovr, Skn
3	73	M	Gastric cancer§	DAD, (PE, PH)	4	Pnc, UGI, Kdn, LGI, Liv, Spl, Hrt, Ubl
4	61	M	Sezary syndrome‡	DAD > IIF, (BP)	N.D.	Pnc
5	67	W	Sepsis	DAD > IIF	N.D.	Pnc, LGI
6	78	M	Urinary bladder cancer‡§, Sepsis	IIF = DAD, (BP)	5	Pnc, UGI, Kdn
7	70	M	Malignant lymphoma‡, NSIP	IIF = DAD	N.D.	Prs
8	65	M	MI§, Mitral insufficiency§†, CRF	IIF = DAD, (BP, PH)	8	UGI
9	68	W	Idiopathic pulmonary fibrosis†	IIF = DAD, (UIP)	4	Pnc, Adr
10	63	W	Lung cancer§	IIF, (BP, OP)	4	None
11	38	M	Chronic myeloid leukemia‡¶	IIF > DAD, (PH)	8	Pnc, Kdn, Adr, Ubl
12	83	M	Idiopathic pulmonary fibrosis†	IIF > DAD, (UIP, PH)	N.D.	N.D.

† Post-immunosuppressive therapy.

‡ Post-adjuvant chemotherapy.

§ Post-operation.

¶ Post-transplantation.

†† CMV-induced lesions with a sign of inequality or equality, according to those predominances, and primary or complicated lesions, shown in parentheses.

‡‡ The period after deterioration or the appearance of respiratory symptoms to death.

Adr, adrenal gland; BP, bronchopneumonia; CRF, chronic renal failure; DAD, diffuse alveolar damage; Gbl, gallbladder; Hrt, heart; IIF, interstitial inflammation/fibrosis; Kdn, kidney; LGI, lower gastrointestinal tract, including small intestine and colon; Liv, liver; MI, myocardial infarction; N.D., not determined; NSIP, non-specific interstitial pneumonia; OP, organizing pneumonia; Ovr, ovary; PE, pulmonary edema; PH, pulmonary hemorrhage; Pnc, pancreas; Prs, prostate; Skn, skin; Spl, spleen; Thy, thyroid gland; Ubl, urinary bladder; UGI, upper gastrointestinal tract, including esophagus, stomach and duodenum; UIP, usual interstitial pneumonia; Utr, uterus; wk, weeks; yr, years.

All 12 patients had underlying primary diseases causing immunosuppression during medical treatment. Systemic CMV infection in multiple organs was observed in all cases except case no. 10. Case no. 12 had regional lung necropsy, although CMV infection in the visceral organs was not determined. Case nos. 1 and 2 had systemic lupus erythematosus with no apparent pulmonary involvement. The pulmonary lesion in case no. 7 was clinically diagnosed as non-specific IP, but was diagnosed as CMVp at autopsy. After deterioration or the appearance of respiratory disease, all patients died of respiratory failure or hypoxia-induced cardiovascular collapse within 1 to 8 weeks, except case nos. 4, 5, 7, and 12 (the durations to death were not reported in the autopsy records) (Table 1).

### Specimens

All autopsied lung materials were fixed in 10% formalin for more than 7 days. At least one tissue block was prepared from the gross lesions in each lobe, and the tissue blocks were embedded in paraffin. In every case, 1 or 2 paraffin-embedded blocks containing the most CMV-infected cells, as shown by CMV IHC, were selected. Additional blocks without CMV-infected cells were prepared in case nos. 9 and 12. A set of 4 µm thick sections was cut for histopathological analyses, including hematoxylin and eosin (HE) and aniline blue staining.

### Primary antibodies

Primary antibodies to cytokeratin (CK) (clone AE1/AE3; Dako, Glostrup, Denmark), CK7 (clone OV-TL 12/30; Dako), surfactant apoprotein A (SP-A) (clone PE10; Dako), vimentin (clone V9; Dako), α-smooth muscle actin (SMA) (clone 1A4; Dako), CD45 (LCA) (clone 2B11 + PD7/26; Dako), CD68 (clone PG-M1; Dako), CMV (clone CCH2 + DDG9; Dako), TGF-β1 (clone TB21; Chemicon, Temecula, CA, USA) and integrin β6 (ITGB6) (clone 442.5C4; Calbiochem, Darmstadt, Germany) were used in this study.

### IHC

Double IHC was performed by combining antibodies for CMV with those for cell type markers or cytokines, using peroxidase (POD)-conjugated universal immuno-enzyme polymer (UIP), anti-mouse solution (Nichirei Biosciences, Tokyo, Japan) and alkaline-phosphatase (ALP)-conjugated UIP, anti-mouse solution. Dewaxed sections were initially incubated in 3% hydrogen peroxide solution at room temperature for 20 min. Antigen retrieval was done according to the manufacturer's instructions. Each antibody reaction was done at 37°C for 1 h. The first round of immunostaining was colored bluish purple with fast blue BB salt (Sigma, St. Louis, MO, USA).^25^ To block cross-reactivity in sequential rounds of immunostaining, the microwave oven heating (MW) method was performed.^26^ The second round of immunostaining was colored red with 3-amino-9-ethyl carbazole (AEC) + substrate-chromogen (DakoCytomation, Carpinteria, CA, USA). The specificity and sensitivity of each marker was verified by single IHC prior to double IHC using POD-conjugated UIP and colored brown with a liquid 3,3′-diaminobenzidine tetrahydrochloride (DAB) substrate chromogen system (DakoCytomation), then counter-stained with hematoxylin.

### Chromogenic *in situ* hybridization (CISH) for whole CMV genome (CISH-CMV) combined with immunostaining of ITGB6

A DNA probe for CISH-CMV, which was derived from a bacterial artificial chromosome (BAC) and encoded 230 kb of the whole genome of human CMV Towne strain (a gift from Dr Fenyong Liu, University of California, Berkeley, USA), was labeled with digoxigenin (DIG)-11-dUTP (Roche Diagnostics, Penzberg, Germany) using a nick translation kit (Roche Diagnostics). The hybridization and washing procedures have been described previously.^27^ Sections were subsequently incubated with POD-conjugated anti-DIG Fab fragments (1:100, Roche Diagnostics) and colored red with AEC+, followed by hematoxylin counter staining.

Combined ITGB6 IHC and CISH-CMV were also performed. The former was preceded with POD-conjugated UIP, and then colored brown with DAB substrate. After MW, CISH was done and colored red with AEC+, and then counterstained with hematoxylin.

### Semi-quantitative reverse transcriptase-polymerase chain reaction (RT-PCR) for *TGF-β1*

Ten slices of 10 µm thick paraffin-embedded tissue sections, which were cut from the same samples used for histology and IHC, were placed into a 1.5 mL tube. Total RNA was extracted using ISOGEN (Nippon gene, Tokyo, Japan), according to the manufacturer's protocol. Following treatment with RNase-free DNase I (Roche Diagnostics) for 15 min at 37°C, RNA was reverse transcribed using the SuperScript II First-Strand Synthesis System (Invitrogen, Carlsbad, CA, USA). Isolated reverse transcribed product (0.5 µg) was used as the RT-PCR template. The primer pairs for RT-PCR were as follows: 5′-AAGATATCGAATTCTCCGAGAAGCGGTAC-3′ and 5′-CGCGGATCCTCCGGTGACATCAAAAGATA-3′ for TGF-β1; 5′-GAAGGTGAAGGTCGGAGTC-3′ and 5′-GAAGATGGTGATGGGATTTC-3′ for glyceraldehyde-3-phosphate dehydrogenase (GAPDH); and 5′-ATGAAGTGTATTGGGCTAACTATGC-3′ and 5′-TTCTCCTAAGTTCATCCTTTTTAGC-3′ for CMV. An initial denaturation step at 95°C for 10 min was followed by 40 cycles of denaturation at 95°C for 30 s, and annealing at 56°C, 58°C, and 60°C for 30 s for TGF-β1, GAPDH, and CMV, respectively. The final elongation step was at 72°C for 10 min. Amplified aliquots were separated on a 2% agarose gel and visualized by ethidium bromide staining.

### *In situ* hybridization (ISH) of *IL-8* messenger RNA (mRNA) and IHC for CMV

To detect *in situ* expression of *IL-8* mRNA, a fragment of *IL-8* complementary DNA, corresponding to nucleotides 1134–1245 (GenBank Accession No. NM_000584), was cloned into the pGEM-T vector (Promega, Madison, WI, USA). Antisense and sense IL-8 riboprobes were prepared with a DIG RNA labeling kit (Roche Diagnostics) using pGEM-T/*IL-8*_1134–1245_ as the template. Hybridization and washing procedures have been previously described.^28^ For signal detection, POD-conjugated anti-DIG Fab fragments were applied, and then colored red with AEC+. The specificity of ISH was demonstrated by parallel hybridization of the sections with sense riboprobes. For double staining by *IL-8* ISH and CMV IHC, completion of the former was followed by MW. CMV IHC was performed using ALP-conjugated UIP and colored bluish purple with fast blue BB salt.

### Cell counting

The number of cells positive for CMV as well as the number of cells double positive for CMV and cellular markers were counted in more than three non-overlapping 5 × 5 mm^2^ fields. The ratio of double positive cells to CMV-positive cells was calculated. Since endothelial cell markers, such as CD31 and factor VIII, tend to lose their immunoreactivity in CMV-infected endothelial cells,^29^^,^^30^ CMV-infected endothelial cells were morphologically identified in double vimentin- and CMV-stained sections and were counted. The average number of CMV-positive cells and the frequency of CMV infection, calculated by the average number of CMV-positive cells per counted area (mm^2^), were also examined.

### Histomorphological evaluation of interstitial fibrosis

Aniline blue stains collagen-deposition areas dark blue compared to the light blue-stained reticulin fibers or basement membrane.^31^ Images of several non-overlapping regions, excluding those with large vessels or bronchi, were taken with a digital camera system (DP70; Olympus, Tokyo, Japan). Each dark blue-stained area was extracted as a two-tone image and the ratio of the stained area to whole image was determined using Adobe Photoshop 7.0 (Adobe Systems Inc., San Jose, CA, USA). The mean aniline blue-positive ratio in each case was used as an indicator of interstitial fibrosis.

## RESULTS

### Histomorphological characteristics of severe CMVp

**Figure 1:**
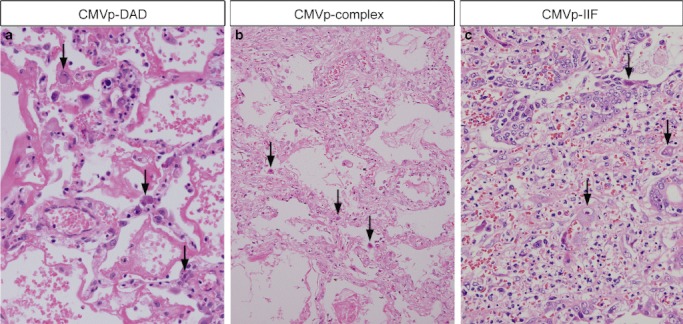
Three major histological patterns of cytomegalovirus (CMV) pneumonia (CMVp). (a) Diffuse alveolar damage (DAD) pattern of CMVp (CMVp-DAD) (case no. 1, x20), (b) intermixed pattern of DAD and interstitial inflammation/fibrosis (IIF) (CMVp-complex) (case no. 7, x10), and (c) predominantly IIF pattern (CMVp-IIF) (case no. 12, x20). Cytomegalic cells (arrows) are observed on the alveolar surface, in the alveolar space, and in the alveolar wall.

Pulmonary lesions are shown in Table 1. CMV-induced pulmonary lesions exhibited diffuse alveolar damage (DAD) and/or interstitial inflammation and fibrosis (IIF). In DAD lesions, hyaline membranes, detached swollen pneumocytes, intra-alveolar exudation, and alveolar wall edema were commonly observed. Cytomegalic cells were frequently seen on the alveolar surface or in the alveolar spaces (Fig. 1a, arrows). In contrast, CMV-induced IIF lesions presented various degrees of interstitial inflammatory infiltrates and fibrous thickening as well as reactive alveolar epithelial proliferation. Cytomegalic cells were observed not only on the alveolar surface, but also in the stroma (Fig. 1b,c, arrows).

Three cases (case nos. 1–3) had DAD with negligible IIF (CMVp-DAD) (Fig. 1a) and six cases (case nos. 4–9) had DAD and IIF with variable severity in a complex pattern (CMVp-complex) (Fig. 1b). In the remaining three cases (case nos. 10–12), IIF was predominant (CMVp-IIF) (Fig. 1c), although minor DAD patterns were also seen in case nos. 11 and 12. All DAD lesions had acute stage characteristics with hyaline membrane formation, except case no. 3, in which foci of intra-alveolar fibrosis, regarded as an organizing stage of DAD, were also observed.

### CMV-infected cell types and CMVp patterns

**Figure 2:**
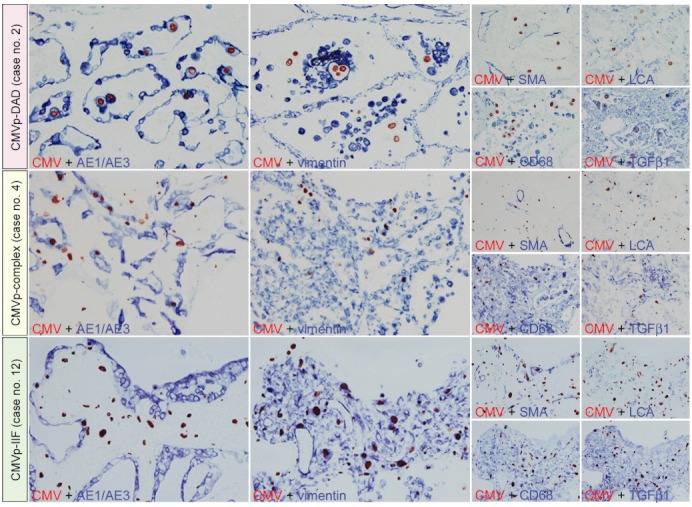
Double immunohistochemistry (IHC) of cytomegalovirus (CMV, red) and each cellular marker (blue) in representative cases of CMV pneumonia (CMVp)-diffuse alveolar damage (DAD) (case no. 2), CMVp-complex (case no. 4), and CMVp- interstitial inflammation/fibrosis (IIF) (case no. 12).

Numerous CMV antigen-positive cells, many more than the number of cytomegalic cells, were found by IHC. These CMV-positive cells were doubly labeled with epithelial, mesenchymal, or leukocyte markers by IHC without cross-reactivity (Fig. 2), except for endothelial cell markers CD31 and factor VIII, which only stained the CMV-negative endothelial cells well (data not shown).

**Figure 3:**
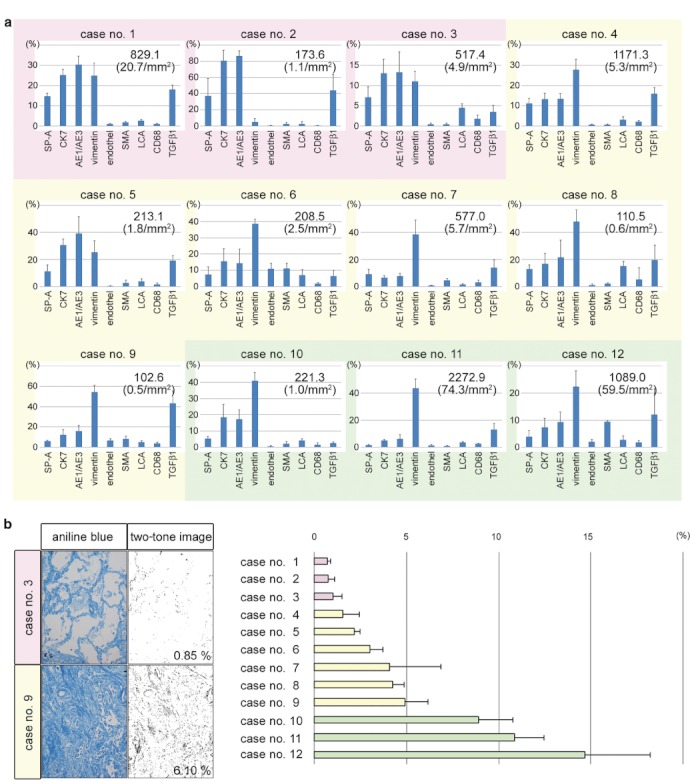
Cytomegalovirus (CMV) tropism and severity of fibrosis among cases. (a) The ratio of doubly positive number for CMV and cellular marker to CMV-positive number in every section with the average number of CMV-positive cells and the frequency of CMV infection shown in parentheses; (b) representative aniline blue stained images and extracted two-tone images with the value of the positive ratio (left panels), and the aniline blue-positive ratio in all cases (right panel). The cases of CMVp-diffuse alveolar damage (DAD) (case nos. 1–3), CMVp-complex (case nos. 4–9), and CMVp-interstitial inflammation/fibrosis (IIF) (case nos. 10–12) are indicated by pink, yellow, and green, respectively.

The results of intrapulmonary CMV tropism by double IHC are shown in Fig. 3a. Although the proportion of CMV-infected cells was generally very small in specific stromal cell types, such as smooth muscle cells, myofibroblasts, leukocytes, macrophages, and endothelial cells, the proportion in vimentin-positive whole stromal cells, including fibroblasts, and cytokeratin-positive epithelial cells, including pneumocytes, was larger and varied among the cases. The major histological patterns of CMVp depended on the proportion of CMV-infected pneumocytes and total stromal cells. In cases with CMVp-DAD, CMV infection was more frequent in pneumocytes than in stromal cells, whereas in CMVp-complex or CMVp-IIF cases, CMV infection was more prevalent in stromal cells, except in case no. 5. Collagen deposition, quantified by aniline blue staining, was less than 3% in CMVp-DAD cases and as high as 14% in CMVp-IIF cases (Fig. 3b); however, the percentage of staining was not strictly proportional to the duration of respiratory symptoms (Table 1) or the ratio of CMV-infected stromal cells (Fig. 3a).

### Expression of TGF-β1 and ITGB6 and presence of the CMV genome associated with CMV propagation

**Figure 4:**
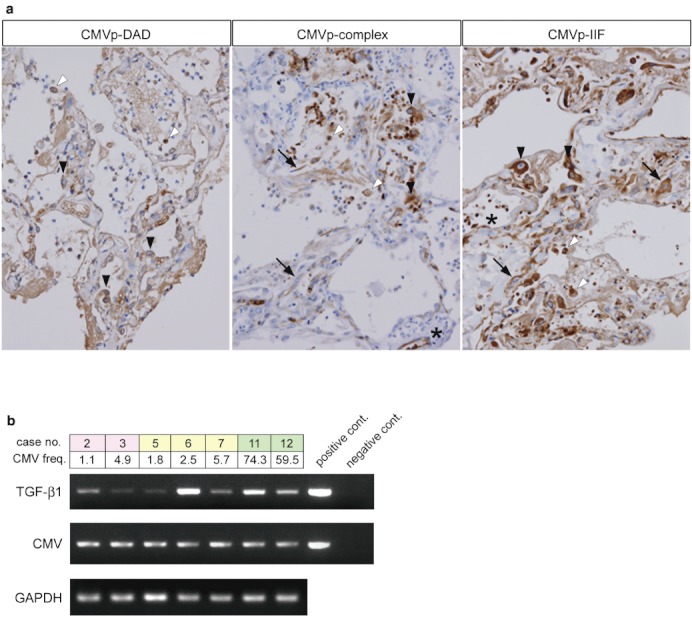
Transforming growth factor (TGF)-β1 expression in immunohistochemistry (IHC) and semi-quantitative reverse transcriptase-polymerase chain reaction (RT-PCR). (a) Representative cases of TGF-β1 IHC of cytomegalovirus pneumonia (CMVp)-diffuse alveolar damage (DAD) (case no. 2) (left panel), CMVp-complex (case no. 5) (middle panel), and CMVp-interstitial inflammation/fibrosis (IIF) (case no. 11) (right panel), the positive findings in the reactive pneumocytes (solid arrowheads), intra-alveolar mononuclear cells (open arrowheads), fibroblasts (arrows), and vascular endothelial cells (asterisks); (b) the findings of semi-quantitative RT-PCR for *TGF-β1*, *CMV*, and *glyceraldehyde-3-phosphate dehydrogenase (GAPDH)* in representative cases of CMVp-DAD (case nos. 2 and 3), CMVp-complex (case nos. 5, 6, and 7), and CMVp-IIF (case nos. 11 and 12) with the frequency of CMV infection (CMV freq.) as described in Figure 3a.

Transforming growth factor-β1 immunoreactivity was observed in various cell types, such as fibroblasts, endothelial cells, reactive pneumocytes, and intra-alveolar mononuclear cells (Fig. 4a). Not all CMV-infected cells were positive for TGF-β1. The percentage of TGF-β1 expressing, CMV-infected cells ranged from 5 to 40% (Fig. 3a), and had no apparent relationship to CMVp pattern. The TGF-β1 staining intensity and distribution also varied among the cases (Fig. 4a). In CMVp-DAD cases, weak to moderate TGF-β1 staining was restricted to swollen pneumocytes and intra-alveolar mononuclear cells (Fig. 4a, left panel). In CMVp-complex and CMVp-IIF cases, TGF-β1 immunoreactivity increased in intensity and was found in both pneumocytes and stromal cells, and stromal cells exhibited heterogeneous and collective staining patterns (Fig. 4a, middle and right panels). Strong TGF-β1 expression was noted in areas of active tissue injury accompanied by CMV infection and inflammatory infiltration.

The expression of *TGF-β1* mRNA, determined by semi-quantitative RT-PCR, was low in representative cases of CMVp-DAD, while expression was relatively high in many CMVp-complex and CMVp-IIF cases (Fig. 4b), which was similar to the TGF-β1 protein expression intensities shown by IHC (Fig. 4a). Expression in CMVp-complex and CMVp-IIF cases was correlated with the CMV infection frequency, except in case no. 6.

**Figure 5:**
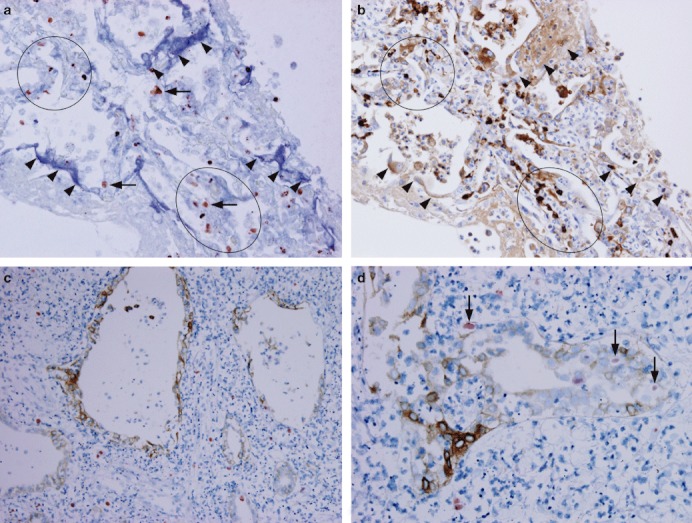
The relationship between cytomegalovirus (CMV) infection/replication and functional expression of transforming growth factor (TGF)-β1, associated with integrin β6 (ITGB6) overexpression. (a) Double immunohistochemistry (IHC) of CMV (red) and ITGB6 (blue) and (b) single IHC of TGF-β1 (brown), examined at the same region of the serial sections (case no. 5). Up-regulation of ITGB6 in the pneumocytes accompanied by TGF-β1 expression (solid arrowheads) as well as colocalization of TGF-β1 expression and CMV antigens (circles) in both CMV pneumonia (CMVp)-complex and CMVp-interstitial inflammation/fibrosis (IIF) cases. (c and d) In double staining of chromogenic *in situ* hybridization for whole CMV genome (CISH-CMV) (red) and ITGB6 IHC (brown) (case no. 12), ITGB6 overexpression area is surrounded by remarkable IIF with increased CISH-positive stromal cells (c, x10). CISH-positive pneumocytes are lacking ITGB6 expression (d, arrows, x40).

Immunohistochemical staining for TGF-β1 and for both ITGB6 and CMV in the serial sections from CMVp-complex or CMVp-IIF cases showed CMV antigens in regions with TGF-β1 expression (Fig. 5a,b, circles), and overexpression of ITGB6 in CMV-negative pneumocytes, but not in CMV-positive pneumocytes (Fig. 5a). In cases of CMVp-DAD, ITGB6 expression was rare, while co-localization of TGF-β1 and CMV was observed in pneumocytes (data not shown).

With CISH-CMV, positive signals were seen, not only in the cytomegalic cells, but also in various types of infected cells with indistinct cytomegaly, since the sensitivity of CISH-CMV is much higher than that of conventional CMV ISH. A large number of CISH-positive cells were often associated with severe DAD and/or IIF lesions. Overexpression of ITGB6 was observed in pneumocytes and accompanied by remarkable proliferative inflammation in the stroma where the number of CISH-positive cells was increased (Fig. 5c). However, as demonstrated by double staining, CISH-CMV-positive pneumocytes significantly lost ITGB6 expression (Fig. 5d, arrows).

### *IL-8* expression and IIF severity

**Figure 6:**
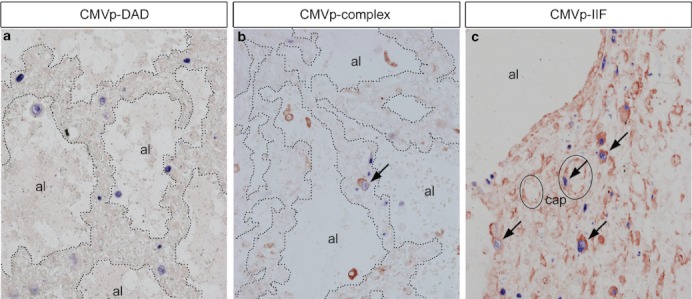
Double staining of *interleukin (IL)-8* ISH (red) and cytomegalovirus (CMV) IHC (blue) in representative cases of CMVp-DAD (a, case no. 1), CMVp-complex (b, case no. 8), and CMVp-interstitial inflammation/fibrosis (IIF) (c, case no. 12); arrows, *IL-8* overexpression in CMV infected cells; al, alveolar space; cap, capillary endothelial cells.

Expression of *IL-8* mRNA by ISH was barely detected in CMVp-DAD cases (Fig. 6a), while focal expression was observed in several cases with CMVp-complex (Fig. 6b). Interestingly, all CMVp-IIF cases showed diffuse *IL-8* up-regulation, and case no. 12 exhibited strong *IL-8* expression (Fig. 6c). Expression of *IL-8* was observed both in pneumocytes and stromal cells, including fibroblastic cells and endothelial cells. Double staining for *IL-8* ISH and CMV IHC suggested that some CMV-infected pneumocytes and stromal cells induced *IL-8* expression (Fig. 6, arrows), particularly in CMVp-IIF, which exhibited remarkable interstitial neutrophilic infiltration.

## DISCUSSION

Here we demonstrated a possible relationship between major CMVp histology and pulmonary viral tropism, namely, CMVp-DAD with epithelial tropism and CMVp-complex or CMVp-IIF with stromal tropism.

**Figure 7:**
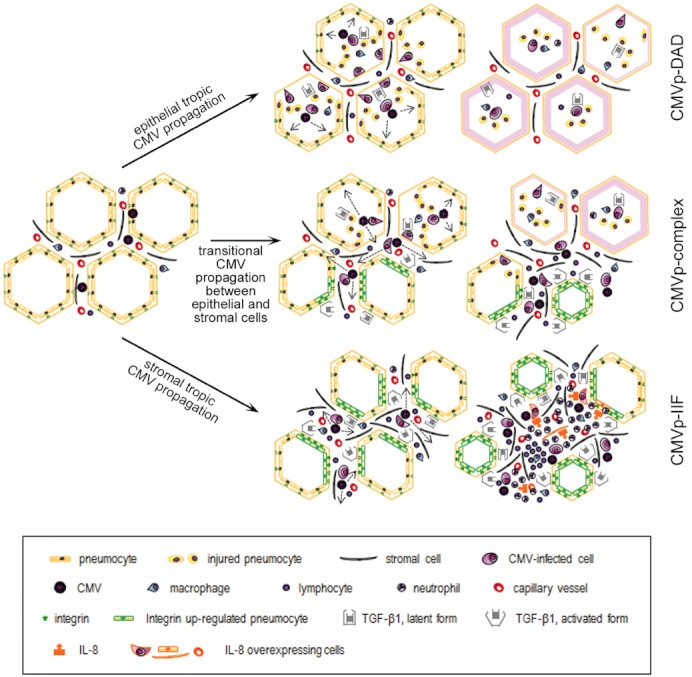
A schema for pathogenesis of cytomegalovirus (CMV) pneumonia (CMVp), focused on the intrapulmonary CMV tropism and expression of transforming growth factor (TGF)-β1, integrin β6 (ITGB6), and interleukin (IL)-8. Epithelial trophic CMV infection causes cellular dysfunction without expression of ITGB6, inhibiting TGF-β1 activation as well as leading to desquamative reaction, which forms diffuse alveolar damage (DAD) lesions (top row). Functional TGF-β1 expression, mediated by up-regulated ITGB6, may promote interstitial chronic proliferative inflammation, according to the CMV infection frequency, that progresses the formation of interstitial inflammation/fibrosis (IIF), especially in the presence of IL-8 overexpression (bottom row). Transitional CMV propagation between pneumocytes and stromal cells causes reciprocal tissue injury of epithelia and stroma, resulted in the complex patterns of DAD and IIF with variable severity (middle row). 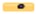, pneumocyte; 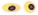, injured pneumocyte; 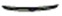, stromal cell; 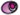, CMV-infected cell; 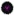, CMV; 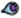, macrophage; 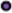, lymphocyte; 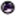, neutrophil; 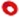, capillary vessel; 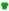, integrin; 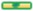, integrin up-regulated pneumocyte; 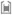, TGF-β1, latent form; 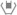, TGF-β1, activated form; 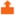, IL-8; 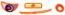, IL-8 overexpressing cell.

The desquamative reactions in CMVp-DAD might be caused by CMV-induced alveolar epithelial injury. Previous reports suggested that CMV-infected pneumocytes tended to lose expression of their functional molecules.^8–10^ Compared with the rates of CK- (AE1/AE3) or CK7-positive CMV-infected cells, the rates of SP-A-positive CMV-infected cells were lower in almost all cases (Fig. 3a). Cell–cell or cell–matrix adhesion molecules, including ITGB6, were also reported to be down-regulated by CMV infection in some permissively infective cells.^32^^,^^33^ Our IHC and CISH analyses confirmed that ITGB6 expression was significantly lost in CMV-infected pneumocytes (Fig. 5d). The decreased adhesion between penumocytes and the basement membrane might result in frequent epithelial detachment, which could be a subsequent source of CMV propagation to other pneumocytes. Finally, plasma exudation, which forms a hyaline membrane on the alveolar surface, causes DAD lesions (Fig. 7).

In addition to its function as an adhesion molecule, ITGB6 has been shown to play an important role in the development of TGF-β1-mediated IP lesions in combination with integrin αv (as a heterodimer).^34^ Transforming growth factor-β1, the majority of which is produced in a latent form,^35^ requires proteolytic enzyme reactions or a conformational change mediated by membrane-bound integrins for activation.^36^ In the absence of ITGB6 expression in CMV-positive pneumocytes, TGF-β1 signal transduction may be insufficient for the development of interstitial lesions, thereby resulting solely in a CMVp-DAD histological pattern, even if increased TGF-β1 expression is present. In case no. 3, which had 4 weeks of symptoms of respiratory disease, the organizing stage of DAD with intra-alveolar fibrous reaction was observed, while IIF lesions were underdeveloped.

Conversely, in CMVp-complex and CMVp-IIF cases, ITGB6 was overexpressed in CMV-negative pneumocytes along with TGF-β1 overexpression (Fig. 5a,b). CISH-CMV revealed a number of CMV-positive cells in the stroma of such ITGB6-overexpressing alveoli (Fig. 5c). Therefore, stromal trophic CMV propagation might promote functional TGF-β1 expression, primarily according to the frequency of CMV infection intensity, and cause persistent interstitial injury, leading to prolonged wound healing and exacerbation of IIF (Fig. 7). The variation in DAD and IIF severity in CMVp-complex may reflect, not a series of DAD lesions, but the reciprocal tissue injury induced by transitional CMV propagation between pneumocytes and stromal cells (Fig. 7).

It is essential to consider host immune status to understand CMV pathology and viral propagation.^37^^,^^38^ Marked systemic CMV involvement in CMVp-DAD cases (Table 1) suggests a profound loss of the immune response against CMV infection. The severe immune-refractoriness may bring about CMV reactivation and rapid propagation in the lung with epithelial tropism, thus leading to CMVp-DAD with slight inflammatory infiltrates despite the alveolar injury.

In contrast, chronic, excessive inflammation, accompanied by *IL-8* overexpression, was noted in cases of heavily infected CMVp-IIF. The CMV genome harbors several functional homologs of cytokine, chemokine, and chemokine receptors.^39^ A transcript of *UL146*, an open reading frame in the CMV genome, is known to act as an IL-8 receptor agonist,^40^ promoting transendothelial migration of the neutrophils and viral transmission through migratory cell–cell microfusion events.^41^ Furthermore, CMV-infected neutrophils are protected from apoptosis and remain in the tissue with enhanced function,^42^ especially in the presence of IL-8.^43^ Hence, CMV can propagate and modulate various host cellular immune functions,^44^^,^^45^ leading to regulatory failure of the host immune system.

In the underlying pathology of IPF in case nos. 9 and 12, IL-8 overexpression may be a factor that exacerbates CMV-induced IIF (Fig. 3b); however, the mechanism underlying induction of *in vivo* IL-8 overexpression remains controversial. Stromal components, such as vascular endothelial cells and myofibroblasts, have been proposed to play a critical role in regulating IPF^46^^,^^47^ and CMVp.^24^ Endothelial cells infected with CMV were found in the present CMVp cases, as in the previous reports;^6^^,^^48^ however, the ratio of positive cells was typically lower (Fig. 3a), and are not likely to be a major regulator of local CMVp lesion formation. Infection with CMV in SMA-positive cells was mostly not found in myofibroblasts, but was observed in pre-existing peribronchiolar or perivascular smooth muscle cells (Fig. 2). As to the concept of epithelial mesenchymal transition, i.e., TGF-β1-mediated cellular transformation from epithelial cells to myofibroblasts,^49^ further evidence is required to elucidate its significance in IIF pathogenesis.

In conclusion, the present histopathological and pathogenetic analysis of severe CMVp suggested that CMV tropism between pneumocytes and stromal cells, CMV-induced cytokine expressions, and host immune conditions were crucial in the formation of a variety of CMVp types.
